# Disparities in antenatal care service utilization among food secure and food insecure women in Gombora District, Hadiya zone, south Ethiopia

**DOI:** 10.11604/pamj.2020.37.377.19862

**Published:** 2020-12-24

**Authors:** Getachew Bitiro Kotiso, Desta Erkalo Abame, Tefera Belachew, Meseret Tamrat, Dejene Ermias Mekango

**Affiliations:** 1 Hadiya Zone Misha, District Health Office, Southern Ethiopia, Ethiopia,; 2 Department of Public Health, College of Medicine and Health Sciences, Wachemo University, Hossana, Ethiopia,; 3 Department of Population and Family Health, Institute of Health, Jimma University, Jimma, Ethiopia

**Keywords:** Food security, antenatal care, Gombora, Hadiya, Ethiopia

## Abstract

**Introduction:**

little is known about antenatal care (ANC) utilization difference among food secure and food insecure household pregnant women and factors contributing to inequities in antenatal care use in developing country including Ethiopia. To determine the disparities in the utilization of antenatal care that exists between pregnant women in food secure and food insecure household women.

**Methods:**

a community based comparative cross-sectional study was conducted in Gombora District, Hadiya zone, southern Ethiopia. Data were collected from February 25^th^ to March 25^th^, 2015, using a pre-tested structured questionnaire. Pregnant women were selected by a simple random sampling method. The data were entered using EpiData 3.1 and exported to SPSS version 21 for analysis. Multivariate logistic regression analysis was done to compare antenatal care utilization among food secure and insecure household women at 95% confidence interval (CI). Statistical tests were done at a level of significance of p<0.05.

**Results:**

two hundred sixty-seven (34.5%) of the respondents received at least one antenatal care visit on current pregnancy. Forty-nine-point one percent of food secure and 23.3% of food insecure household women utilized ANC from health professionals. Factors associated with antenatal care utilization included being from a food secure household (adjusted odds ratio [aOR]= 2.54; 95%CI: 1.79-3.59), having attained secondary or higher education (aOR=3.76; 95%CI: 2.32-6.1), good level of knowledge of antenatal care (aOR= 2.42; 95%CI 1.34-4.33) and being from a wealthy household (aOR=2.10; 95% CI: 1.34-3.28). **Conclusion:** this study showed a significant variation in the use of ANC in food secure and food insecure household pregnant women. Interventions to improve ANC utilization should prioritize women from poor socio-economic and low educational background.

## Introduction

Inadequate access and under-utilization of modern healthcare services are major reasons for poor health in developing countries. This inequality in the health and well-being of women in the developing world is a growing concern [1]. Antenatal care is one of the most key indicators for controlling maternal morbidity and mortality. In most of the developing countries, women do not receive appropriate ante-natal care during pregnancy particularly in places where the women health status is poor through counseling on nutrition, birth preparedness, delivery care and family planning options. ANC is a good opportunity for identifying high risk of pregnancy; improve the outcome of pregnancy and birth for both mother and child [1-3].

Ethiopia is one of the countries in sub-Saharan Africa with a markedly the high maternal mortality ratio. More than half a million maternal death related to pregnancy and childbirth could be saved if they were provided with access to antenatal care and skilled attendants at birth as well as appropriate modern technology to deal with emergency obstetric care situations when needed [2]. In most of the developing countries women do not receive appropriate antenatal care during pregnancy [3]. Even though ANC services in Ethiopia were provided free of charge in all government health institutions, only thirty-four percent of pregnant mothers received antenatal care from a skilled provider. In the rural area 63.1% women did not attend ANC at all. This shows very low utilization of antenatal care in national level and rural area [4].

For more than 20 years, Africa has struggled with hunger and food insecurity. Food security is a situation “when all people, at all times, have physical, social and economic access to sufficient, safe and nutritious food that meets their dietary needs and food preferences for an active and healthy life” [5]. Despite the general worldwide reduction in food insecurity, Africa´s food security and nutrition situation is growing worse [6]. Globally, certain groups of people including women of reproductive age are more vulnerable to food insecurity than others [7]. According to Food and Agriculture Organization (FAO) 2009 report households during economic crises used different coping strategy by changing dietary habit as eating fewer meals, eating lower-quality foods, shifting health expense and reduced use of health services in a poor country of Armenia, Bangladesh and Zambia and in Ghana [8]. In a study conducted in Zimbabwe, 2841 (32.8%) pregnant women were food insecure and 1518 (17.5%) pregnant women were food insecure with hunger. Also, 62% food insecure (FI) women with hunger were more likely to never have attended ANC compared with food secure women [9]. Food insecurity has played a large role in slowing progress on the health millennium development goals (MDGs), especially for children and mothers. About half of pregnant women in developing countries suffer from anemia. Malnourished pregnant women are more likely to give birth to underweight babies, who are more likely to die before their fifth birthday [8,10].

It has been evident that in food insecure situations health care services tend to be forfeited as people make gaining access to food their primary activity and use different coping strategies. Although ANC is a free service, there are indirect and opportunity costs that could deter pregnant women from using the service in food insecure situations. Demographic and socio-economic characteristics influence women´s ANC utilization. These include women´s education, husband´s education, pregnancy intention, age of women at pregnancy and parity, family size and maternal occupation, husbands occupation, wealth index, knowledge on timing (right gestational age), frequency of ANC and interval between visits, were predictors for utilization of ANC. However, all reviewed pieces of literature were not showed about the association between antenatal care utilization and the food security status of pregnant women. But a few studies showed that food insecurity affects health care utilization. However, there is no study documented the association between food insecurity and ANC service use in Ethiopia, particularly in the study area. Therefore, this study might contribute evidence based facts to fill the gaps identified.

## Methods

**Study setting and design:** the community based comparative cross sectional study was conducted in Gombora District, southern nation nationality and people region, Ethiopia. Gombora, one of the rural districts in the Hadiya zone, is located in the southern region of Ethiopia. Gombora District is one of 17 districts found in the Hadiya zone and its capital town which is Hebicho town. The district located at 32 km southwest from the zonal capital town of Hosanna. The data collection period was from Feb 25^th^ to March 25^th^, 2015.

**Study population and sampling:** the study subjects were 796 pregnant mothers from 12 kebeles of Gombora selected by simple random sampling technique. Sampling frame was taken from health posts family folder. Then based on the population size, proportional allocation was done. Finally, households with pregnant women were selected by randomly using research randomizer online and 796 pregnant women interviewed in their home. The sample size was calculated by using Epi info version 7 for two population proportion assumptions; the proportion of ANC users among a proportion of food secure women=41% and proportion of ANC users among proportion of food insecure women=29% (mini Ethiopian demographic health survey (EDHS, 2014)) power 80%, margin of error of 0.05 with two-sided and, 95% confidence interval to reach 528 pregnant mothers. After the finite population correction, 10% non-response rate and 1.55 design effect were considered to obtain the final sample size 796.

**Data collection:** the questionnaire was prepared in English, translated to the local language Hadiyyisa and back-translated to English separately by two individuals to ensure consistency. The data were collected using a pretested questionnaire. The questionnaires were prepared to assess socio-demographic characteristics, utilization of ANC and food insecurity. The data were collected with eight data collectors and three supervisors, who were qualified with a diploma in nursing and bachelor of Science (BSc). in public health. The data collectors and the supervisors were trained for two days on the questionnaire; approach to the interviewees, details of interviewing techniques then interviewed the respondents in Hadiyyisa language. Food security exists when all people, at all times, have physical and economic access to sufficient safe and nutritious food that meets their dietary needs and food preferences for an active and healthy life (World Food Summit, 1996). Characteristics of the household food insecurity were assessed by using the standard nine question item Household Food Insecurity Access Scale (HFIAS) questionnaire by food and nutrition technical assistance three (10). All “yes” responses were coded as one (1) and “no” responses were coded as zero (0) and then for each question frequency was asked and coded as a) rarely (once or twice in the past four weeks), b) sometimes (three to ten times in the past four weeks), c) often (more than ten times in the past four weeks) responses we resumed to produce an index of household food insecurity. The index had an adequate internal consistency (Crone Bach´s alpha = 0.80) and calculate the household food insecurity access category for each household. Finally food secure and mildly food insecure were grouped under food secure and moderately food insecure access and severely food insecure access households were grouped as food insecure. Data collection was supervised and data checked for consistency and completeness. Incomplete and unclear questionnaires were returned to interviewers to be completed.

**Statistical analysis:** the data were entered in double, checked for missing values and outliers and analyzed using SPSS for windows version 21.0. Means and proportions were compared by household food security status using Chi-square tests. First, bivariate analyses were carried out and results were presented using proportions, means and crude odds ratios. Hence variables with p-value<0.25 in the bivariate analysis were taken as candidates for multivariate analysis. Then the multivariable logistic regression model was performed to isolate the independent effect of food insecurity on ANC attendance after adjusting for potential confounder. The results were presented using adjusted odds ratios and 95% confidence intervals. Statistical significance will be declared at p-value<0.05.

**Ethical considerations:** ethical clearance was obtained from, Jimma University, Institute of Health Sciences the Institutional Health Research Ethics Review Committee. A formal letter of permission and support were written to the Gombora District health office. All the study participants were informed about the purpose of the study, their right to refuse and they were also told that the information obtained from them would be treated with complete confidentiality and do not cause any harm except losing a few times. Then, written and signed informed voluntary consent was obtained from all study participants before data collection.

## Results

The overall response rate of the study subject was 97%. Six hundred thirty-three (81.8%) of the respondents were in the age group of 25-34 with the median age of 29 ± 5.48 years. Of 774 pregnant women, 262 (34%) of them were found in the middle tertiles of the household´s wealth index, 257 (33.2%) in the lowest tertiles and the highest/third tertiles 225(32.8%). From the food insecure household women 183 (41.1%), 153 (34.4%) and 109 (24.5%) were composed at lowest, middle and third wealth index groups respectively. Among the pregnant women, 438 (56.6%) were from food insecure households and 336 (43.4%) were from food secure households. Among the food insecure household women, 53 (12%) were mildly food insecure, 205 (46.8%) were moderately food insecure and 180 (41%) were severely food insecure households (Table 1).

**Table 1:** sociodemographic characteristics of the women among interviewed women in Gombora District, Hadiya zone, south Ethiopia, 2015

Characteristics	Count(percent)
**Ethnicity**	
Hadiya	748(96.9)
Kembata	5(0.6)
Gurage	3(0.4)
Silite	3(0.4)
Others*	15(1.9)
**Mothers current occupation**	
House wife	742(95.9)
Civil servant	13(1.7)
Merchant	12(1.6)
Farmer	4(0.5)
Others**	3(0.4)
**Religion of mother**	
Protestant	749(96.8)
Catholic	25(3.2)
**Marital status**	
Married	768(99.2)
Divorced	4(0.5)
Never married	2(0.3)
**Mothers education**	
No education	350(45.2)
Primary (1-8)	280(36.2)
Secondary (9-12)+	144(18.6)
**Husbands education**	
No education	175(22.6)
Primary (1-8)	491(63.4)
Secondary (9-12)+	76(9.8)
Above secondary	32(4.1)
**Wealth index**	
Lowest	257(33.2)
Middle	263(34)
Highest/third	254(32.8)
**Food security status**	
Food secure	336(43.4%)
Food insecure	438(56.6%)

* Fuga ethnic groups; **preacher, daily laborer, carpenters, pottery worker, tired from army force

**ANC utilization:** among the study participant, 237 (30.6%) had at least one antenatal visit during this pregnancy from skilled health professionals (doctors, nurses, midwife) and 30 (3.9%) of the mothers were received ANC care from health extension workers (HEW), while 507 (66.5%) had none. Only 12% (n=32) had the .recommended 4 or more ANC visit from skilled professionals. Eighty-eight percent (88%) of women have not done their first ANC visit according to World Health Organization (WHO) recommendation (less than 4 months of pregnancy). The mean duration of pregnancy at the first visit is 6 months (Table 2).

**Table 2:** antenatal care utilization from skilled professionals and health extension workers in Gombora District, Hadiya zone, south Ethiopia 2015

ANC utilization	Number	Percent
At least one ANC from skilled professionals	237	30.6%
At least one ANC from health extension workers	30	3.9%
Four (4) and more ANC	32	12.0%
No ANC from skilled professionals and health extension workers	507	66.5%

**Prevalence of ANC utilization among food secure and food insecure:** as Table 3 shows the household food security and utilization of ANC, 165 (49.1%) of the food secure women attended ANC service during the current pregnancy. However, only 102 (23.3 %) of food insecure women had attended ANC service. One hundred and seventy-one (50.9%) from food secure households and 336 (76.7%) from food insecure households were not utilized ANC at all. From moderate food insecure household women (N=205) only 41 (20%) and from severely food insecure household women (N=180) only 36 (20%) attended ANC service.

**Table 3:** percentage distribution of antenatal care utilization from skilled health workers among food secure and insecure household women in Gombora District, Hadiya zone, south Ethiopia 2015

Food security state	ANC			
	Yes	Percent	No	Percent
Food secure	165	49.10%	171	50.90%
Food insecure	102	23.30%	336	76.70%

**Reason for attending ANC service among food secure and food insecure women:** from those who attended ANC service 37.6%, 53.9%, 35.8%, 34.5% from food secure and 46%, 41.2%, 35.3% and 23.5% of food insecure household mothers visited a health institution because of fear of problem, to take the vaccination, to have a healthy child and to take iron for anemia prevention respectively, while 61 (22.8%) of the mothers visited a facility for a regular checkup (Table 4).

**Table 4:** reason for attending antenatal care among food secure and food insecure women in Gombora District, Hadiya zone, south Ethiopia 2015

Reason for attending ANC service	Food secure		Food insecure	
	Number	%	Number	%
Fear of problem	126	37.6%	202	46.1%
To take vaccination	181	53.9%	180	41.2%
To have healthy child	120	35.8%	155	35.3%
To take iron for anemia prevention	116	34.5%	103	23.5%
To start regular check up	83	24.8%	86	19.6%
Felt discomfort/illness	61	18.2%	103	23.5%
To confirm pregnancy	35	10.3%	60	13.7%

**Factors affecting the use of ANC services:** as shown in Table 5, factors associated with antenatal care utilization included being from a food secure household (adjusted odds ratio [aOR]= 2.54; 95%CI: 1.79-3.59), having attained secondary or higher education (aOR=3.76; 95%CI: 2.32-6.1), good level of knowledge of antenatal care (aOR= 2.42; 95%CI 1.34-4.33) and being from a wealthy household (aOR= 2.10; 95% CI: 1.34-3.28).

**Table 5:** association of antenatal care utilization and household food insecurity status and other factors in Gombora District, Hadiya zone, south Ethiopia, 2015 (n=774)

Variables	ANC		COR(95 % CI)	AOR(95 % CI)
	Yes	No		
**Household food security**				
Food insecure	102	336	1	1
Food secure	165	171	3.17(2.33, 4.32)	2.53(1.79, 3.59)**
**Education of mother**				
No education	68	282	1	1
Primary education	119	161	3.06(2.14, 4.37)	2.52(1.70, 3.75)**
Secondary and above	80	64	5.18(3.39, 7.90)	3.76(2.32, 6.09)**
**Knowledge on ANC**				
Poor knowledge	19	95	1	1
Good knowledge	248	412	3.01(1.79, 5.04)	2.41(1.31, 4.33)*
**Wealth index**				
Lowest	57	200	1	1
Middle	83	179	1.62(1.09, 2.41)	1.31(.85, 2.02)
Highest	127	128	3.48(2.37, 5.10)	2.10(1.34, 3.28)**
**Pregnancy intention**				
No	80	218	1	1
Yes	187	289	1.76(1.28, 2.41)	1.54(1.04, 2.28)*
**Age of the mother**				
Age 15-24	53	38	1.04(.64, 1.69)	0.91(0.44, 1.90)
Age 25-34	162	331	0.83(0.56, 1.23)	0.71(0.43, 1.18)
Age above 35 years	52	89	1	1
**Number of children**				
No child	36	51	1.89(1.04, 3.34)	0.517(0.18, 1.141)
One child	34	79	1.13(0.64, 2.00)	0.53(0.24, 1.17)
2-4 child	163	287	1.50(0.96, 2.33)	1.05(0.60, 1.85)
>=5 child	34	90	1	1

** p-value<=0.001; * p-value<0.05

## Discussion

In this study, almost seven in ten women did not attend antenatal care during their current pregnancy. Even among the users of ANC, only 12% of women made their first antenatal visit according to WHO recommendation (less than four months of pregnancy) and only 12% of women attended four and more antenatal care services during their current pregnancy. Also, 88% of the women started using antenatal care services later than the recommended time by WHO. They started mostly in their second and third trimester of pregnancy, which is in agreement with the findings of other studies in Ethiopia [11-16]. The reason for this low ANC4 attendance might be late initiation/booking, lack of knowledge on the correct time when to start ANC and on the advantage of early initiation of ANC. The overall utilization of ANC on this study from skilled health worker was 30.6%, which is line with the report from the south region (35.9%) and with the rural coverage report of EDHS-2016 (35%) but lower from Yem special district, southwestern Ethiopia study (40%) of mothers had ANC visit [17,18].

In the current study, we found that more than half (56.6%) of women were living in food insecure households in the month during the survey. ANC attendance was significantly lower (23%) among food insecure pregnant women when compared with food secure household pregnant women ANC utilization which is 49%. Only 20% of women from moderately food insecure household and severely food insecure household women attended ANC service. Pregnant women from food secure household were more likely to use antenatal care compared to those from food insecure household, which is consistent with the study done in Zimbabwe [9]. Other studies were done in Armenia and America also revealed that food insecurity reduces health service utilization [8,19]. The reason for the low utilization of antenatal care among food insecure household women might be giving their attention to the coping strategy for food rather than going to seek care from the health institutions. it might also be opportunistic cost like travel time and due to food shortage they skipped meals or reduce meal size then they become weak to walk to health facility on foot to receive ANC.

Wealth is one of the determinant factors affecting antenatal care service utilization. Those women living in the highest wealth index were utilizing antenatal care service more likely than counterparts, which is supported by the study done in Ethiopia and India [15,20,21]. The possible reason might be women didn´t go to spend the entire day at the health center for their check-ups because of the indirect cost (travel time) and they may go to the market to earn a daily living. The observed association between educational status and ANC utilization is consistent with other reports [3,11-16,22-26]. Hence, education might enhance female autonomy, the ability to make decisions about their health and the increased chance of accessing information.

Another predictor of ANC utilization was the knowledge of mothers, which is supported by several studies done in Ethiopia [11,16]. As mothers have better knowledge, this may increase understanding and acceptance of the ANC service utilization further. Intended pregnancy is encouraged to attend ANC services, which is in line with a study conducted in Yem special district and Mekelle, Ethiopia [18,27]. The possible explanation is that those mothers who had intended pregnancy might have a good awareness about the advantages of antenatal care services utilization.

## Conclusion

This study showed significant variations in the utilization of antenatal care among food secure and food insecure household pregnant women. Also, this study found that low utilization of ANC services in the study area. This study also identified several factors correlated with ANC utilizations. Levels of education, food insecurity, unintended pregnancy and knowledge and wealth index were potent predictors of ANC utilization. The government should give emphasize to those women living in food insecure household to achieve the planned maternal health goals. It must simultaneously help women and providers understand the determinants of ANC utilization at the facility, community and individual levels. Also, health care providers should focus on behavioral change communications (BCC) at grass-root levels.

### What is known about this topic

- Antenatal care is a crucial indicator for controlling maternal morbidity and mortality even though the situation of antenatal care service utilization is low in developing countries;
- Studies have been undertaken in various countries on the factors related to antenatal care service utilization and these studies reviewing antenatal care service utilization have used various indicators.

### What this study adds

- We found the disparities in antenatal care utilization among food secure and food insecure household pregnant women; thus, there was significant variation in the use of ANC in food secure and food insecure household pregnant women;
- This study showed the association between food insecurity and antenatal care service use in Ethiopia, particularly in the study area;
- There was low utilization of antenatal care services particularly for food insecure household pregnant women in the Gombora District, southern Ethiopia.
